# Age-related changes in tricuspid inflow: comparison between phase contrast MR imaging and Doppler echocardiography

**DOI:** 10.1186/1532-429X-13-S1-P350

**Published:** 2011-02-02

**Authors:** Stephanie Clement-Guinaudeau, Emilie Bollache, Muriel Lefort, Magalie Ladouceur, Ludivine Perdrix, Alban Redheuil, Nadjia Kachenoura, Elie Mousseaux

**Affiliations:** 1 Radiology Department HEGP, Paris, France; 2 INSERM U678 Paris 6 University, Paris, France; 3 Cardiology Department HEGP, Paris, France

## Objectives

To compare phase contrast magnetic resonance (PCMR) evaluation of tricuspid inflow against echocardiographic measurements and to assess age-related changes in right ventricular (RV) diastolic function evaluated by both techniques.

## Background

Several echocardiographic studies demonstrated the age-related changes in RV diastolic function. An MRI study supported these findings using SSFP sequences but to our knowledge, there is no such evaluation using PCMR images. Although blood flow PCMR imaging is less operator-dependant than echocardiography, which is strongly conditioned by the transducer orientation, post-processing of these data remains time-consuming and precludes clinical applications. Accordingly, the primary goal of this study was to develop a reproducible and accurate method to assess RV diastolic function.

## Methods

We studied 55 healthy subjects (26 men, age: 42±17 years ranged between 13 and 79 years) who had Doppler echocardiography (GE Vivid7) and PCMR imaging (GE 1.5T) on the same day. The study protocol was approved by the institutional review board and informed consent was obtained from all participants. The previously acquired long axis views were used for positioning a retrospectively ECG-gated PC pulse sequences, in a plane perpendicular to the tricuspid inflow at the level of the tips of the opened tricuspid leaflets. PC images were analyzed using a custom software previously designed to semi-automatically assess transmitral flow. This analysis allowed extracting RV diastolic parameters such as: the peak filling rate (Ef, ml/s) and the peak atrial filling rate (Af, ml/s) and the Ef/Af ratio. These parameters were compared to the known echocardiographic parameters (early and late peak velocities (E, A, cm/s) and the E/A ratio). Inter-operators variability in PCMR measurements was calculated as the absolute difference of the repeated measurements in the percentage of their mean.

## Results

**Figure 1:**
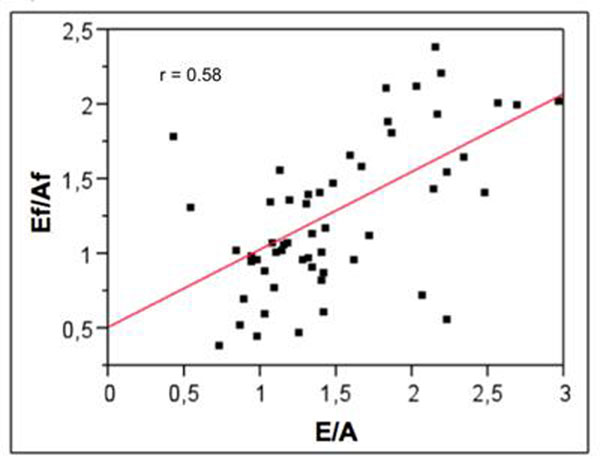
Correlation between E/A and Ef/Af ratios

**Figure 2:**
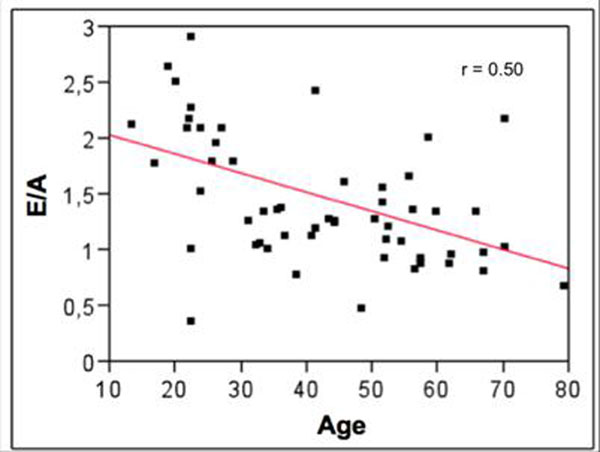
Correlation between age and E/A ratio

**Figure 3:**
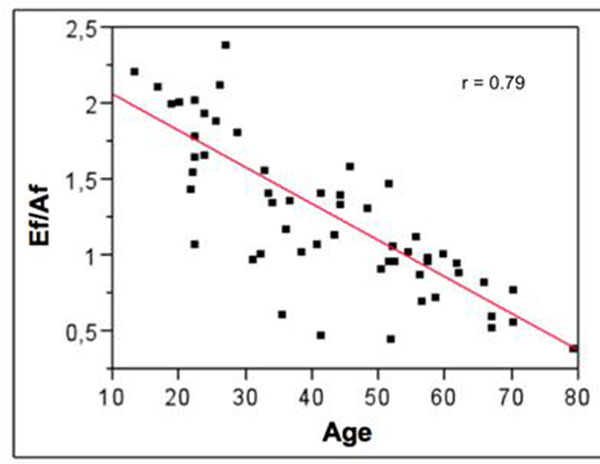
Correlation between age and Ef/Af ratio

Low inter-operators variability in PCMR measurements was found (2.50±2,99%). Comparison between PCMR early to late peak flow rate ratio (Ef/Af) and echocardiographic early to late peak velocity ratio (E/A) resulted in a fair correlation (r=0.58; p=0.0001; Figure 1). However, a stronger correlation with aging was found when considering the PCMR parameter. Indeed, correlation between age and the echocardiographic ratio (Figure 2) resulted in r=0.5 (p<0.001) while it resulted in r=0.79 (p<0.001 ) for the PCMR ratio (Figure 3).

## Conclusion

Fast and reproducible evaluation of tricuspid inflow from PCMR images is possible. Although the diastolic parameters obtained from PCMR images are only fairly correlated with echocardiographic measurements, a stronger relation with aging was found while using PCMR than echocardiographic data.

